# Audio-Motor Training Enhances Auditory and Proprioceptive Functions in the Blind Adult

**DOI:** 10.3389/fnins.2019.01272

**Published:** 2019-11-22

**Authors:** Anna Vera Cuppone, Giulia Cappagli, Monica Gori

**Affiliations:** ^1^Unit for Visually Impaired People, Fondazione Istituto Italiano di Tecnologia, Genoa, Italy; ^2^IRCSS Fondazione Istituto Neurologico C. Mondino, Pavia, Italy

**Keywords:** blindness, training, plasticity, audition, proprioception

## Abstract

Several reports indicate that spatial perception in blind individuals can be impaired as the lack of visual experience severely affects the development of multisensory spatial correspondences. Despite the growing interest in the development of technological devices to support blind people in their daily lives, very few studies have assessed the benefit of interventions that help to refine sensorimotor perception. In the present study, we directly investigated the impact of a short audio-motor training on auditory and proprioceptive spatial perception in blind individuals. Our findings indicate that auditory and proprioceptive spatial capabilities can be enhanced through interventions designed to foster sensorimotor perception in the form of audio-motor correspondences, demonstrating the importance of the early introduction of sensorimotor training in therapeutic intervention for blind individuals.

## Introduction

Recent evidence suggests that some spatial capabilities in blind individuals may be delayed or compromised (Pasqualotto and Proulx, 2012; Gori et al., 2013; Voss et al., 2015; Cuturi et al., 2016). This has been associated with the reduced accessibility to multisensory experiences caused by the lack of vision during the first years of life when plasticity is maximal and the critical period for the development of spatial representation can develop (Putzar et al., 2007; Cappagli et al., 2017b). Impairments of spatial representation is not limited to tactile and auditory perception (Röder et al., 2004; Gori et al., 2013; Finocchietti et al., 2015a; Vercillo et al., 2016), but it also extends to proprioception (Rossetti et al., 1996; Gaunet and Rossetti, 2006; Fiehler et al., 2009; Cappagli et al., 2017a). Given the risk of developing spatial deficits due to the lack of vision, specific training to improve spatial skills would be fundamental for individuals with a visual disability.

Despite their potential usefulness for rehabilitation purposes, the benefit of interventions based on sensorimotor contingencies, such as audio-motor correspondence, has been barely studied in the blind population. Conversely, the use of auditory information coupled with visual or motor feedback has been mainly studied in robotic therapy systems to motivate or guide patients in the execution of performance tasks (Maulucci and Eckhouse, 2001; Robertson et al., 2009), generally reporting positive outcomes (Sigrist et al., 2013). Several works have demonstrated that the use of audition to complement or substitute visual information provides users with additional feedback of their own movements (Bevilacqua et al., 2016; Cappagli et al., 2019). For instance, it has been shown that when coupled with visual feedback, continuous task-related audio information can improve motor performance and facilitate the learning of a novel visuomotor perturbation, indicating that auditory augmentation of visual feedback can enhance upper limb sensorimotor learning (Rosati et al., 2012). Auditory feedback can also substitute visual feedback for specific tasks, e.g., it can convey information to estimate the curvature of a virtual shape when visual feedback is temporarily removed (Boyer et al., 2015), suggesting that specific stimulus features can be translated from one modality to another. These results demonstrate that interventions based on meaningful multisensory correspondences can augment sensorimotor learning.

To date, research investigating the effect of auditory information to improve spatial perception in the case of blindness mainly focused on the evaluation of sensory substitution devices which tend to substitute vision with audition without specifically providing sensorimotor correspondences (Amedi et al., 2007; Auvray and Myin, 2009; Chebat et al., 2011; Striem-Amit et al., 2012). Only few studies assessed the effects of pure audio-motor training on spatial cognition in the blind, reporting positive outcomes in the case of training with an external auditory sound source that provides sonorous feedback of body movements (Aggius-Vella et al., 2017; Cappagli et al., 2017b, 2019; Finocchietti et al., 2017). In all these studies, the auditory feedback was actively generated by the individual through his own body movements thus spatial information emerged from the coupling of sensorimotor contingencies. For this reason, the training was less demanding compared to the training required for sensory substitution devices, since it only required individuals to naturally associate auditory and motor information coming from their body without learning codification rules requested by an external substitution device. These studies demonstrated that an audio-motor training has a positive effect on auditory and proprioceptive spatial perception in blind children, but they did not tested if the same effect is visible for blind adults, which has been shown to be impaired from an early age for proprioceptive functions (Rossetti et al., 1996; Gaunet and Rossetti, 2006; Cappagli et al., 2017a). We recently showed that sighted people improve their proprioceptive spatial abilities after an audio-motor training (Cuppone et al., 2018), highlighting substantial differences between training modalities and feedback types, but no studies to date have explored if blind individuals show similar enhancement in their proprioceptive functions.

For this reason, in the present study, we assessed the impact of an audio-motor training on spatial capabilities in visually impaired individuals, to test whether experiencing an auditory feedback of body movements can refine spatial mapping across multiple domains, namely auditory and proprioceptive domains. With this aim, we compared auditory and proprioceptive localization accuracy before and after a short sensorimotor training in which passive movements of the dominant arm of participants were enriched with a continuous or discrete audio feedback that creates a spatial audio-motor association. To assess the presence of generalization effects, we examined whether auditory and proprioceptive functions were improved also on the untrained side of the body, namely the non-dominant arm.

## Materials and Methods

### Participants

The study involved 16 participants with no known neuromuscular disorders and naïve to the task. The participants were divided into two groups: a sighted training group (*n* = 7; age: 32 ± 4) and a blind training group (*n* = 9; age: 41 ± 15) who performed the same training. A *t*-test confirmed that the two groups did not differ in terms of chronological age [*t*(14) = −1.54, *p* > 0.05]. Blind participants have been considered as *early blind* since the loss of vision occurred within the third year of age despite the fact that diagnosis was known at birth. The clinical details of the early blind participants are reported in Table 1. The research conformed to the ethical standards laid down in the 1964 Declaration of Helsinki and was approved by the local ethics committee (ASL3 Ligure). Each participant signed an informed consent form conforming to these guidelines.

**TABLE 1:** Clinical details of the group of visually impaired participants.

**Subject**	**Age range**	**Residual vision**	**Pathology**
1	55–60	^∗^	Uveitis
2	20–25	^∧^	Leber’s congenital amaurosis
3	25–30	^∧^	Retinopathy of Prematurity
4	20–25	^∧^	Congenital cataract
5	55–60	^∧^	Congenital glaucoma
6	25–30	^∧^	Retinopathy of Prematurity
7	60–65	^∧^	Atrophy of the eyeball
8	40–45	^∧^	Congenital glaucoma
9	50–55	^∗^	Retinitis pigmentosa

*The table shows for each subject the information related to age, residual vision and pathology. ^∧^No residual vision, ^∗^residual vision (light and shadow).*

### Procedure

The protocol consisted of one pre-test and one post-test session (*Assessment phase*) where two different aspects of spatial cognition were investigated (auditory and proprioceptive localization) and one training session (*Training phase*) performed between the pre-test and post-test sessions. The first assessment task is related to the auditory domain and investigated participants’ ability to localize sounds in space (*Reaching of auditory cue task*) while the second assessment task is related to the proprioceptive domain and investigated the participants’ ability to reproduce a position in space (*Joint position matching task*). The tasks included in the *Assessment phase* have been already presented in Cuppone et al. (2018). During both tasks, all participants were blindfolded and each participant performed the assessment tests both with the dominant and non-dominant arms. During the *Training phase*, the trained arm was always the dominant one. This allowed us to assess whether the training effect generalizes to the untrained (non-dominant) arm.

### Assessment Phase

**FIGURE 1:**
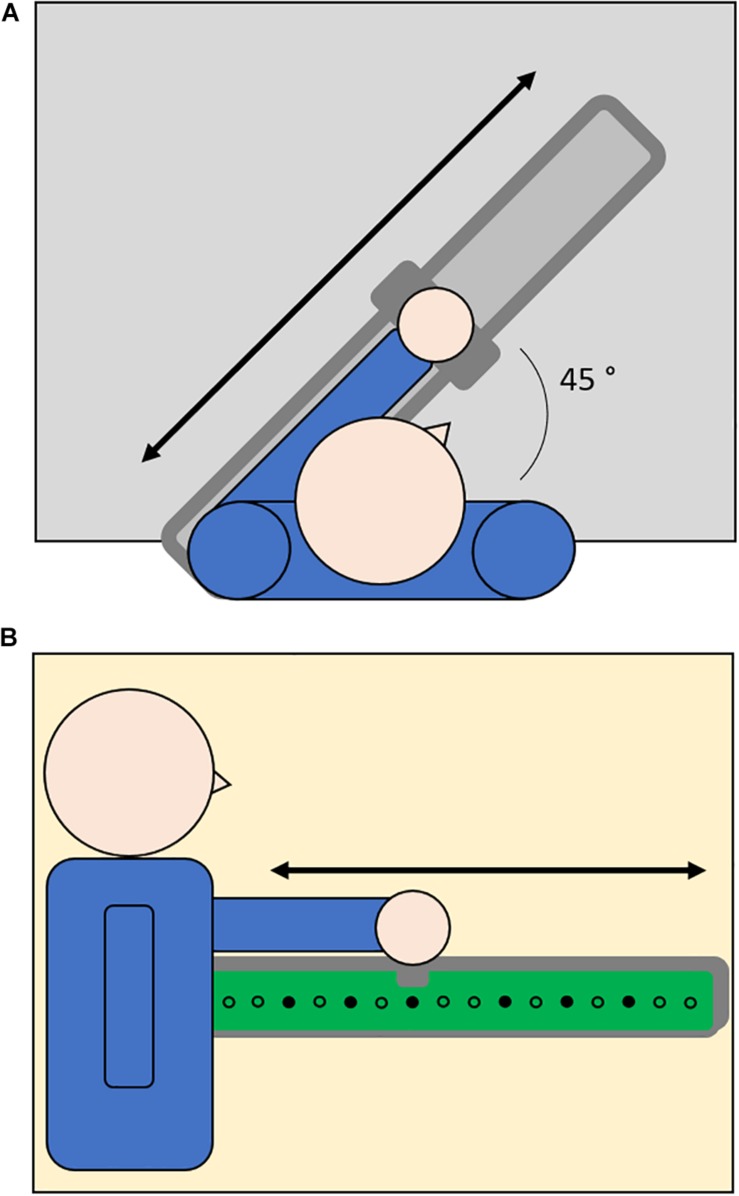
Top-view **(A)** and side-view **(B)** of setup configuration. Participants were seated at a table and their dominant or non-dominant arm was positioned on a metallic rail (light gray, 1A) placed above an array of audio speakers (dots, 1B) mounted on a tactile surface that can register the position of the contact (green surface, 1B). To perform the task, participants were asked to hold a handle to slide over the metallic rail and indicate the position of auditory (Reaching of Auditory Cue Task) or proprioceptive (Joint Position Matching Task) targets (filled dots).

The setup shown in Figure 1 utilized a set of 16 loudspeakers embedded in an array covered by tactile sensors (1 cm ⋅ 1 cm) that can register the position of the contact and provide accurate information about spatial errors. The setup was fixed on the desk in front of the participants along a line inclined with an angle of 45° with respect to the frontal axis of the human body (Figure 1A). The center of the setup was kept 20 cm far from the center of the body in order to allow participants to easily reach farther positions. The participants held a handle to slide on a metallic rail positioned on the setup. The system was controlled by a workstation and the software environment was implemented in Matlab. The serial communication between the workstation and the loudspeakers was bidirectional and it allowed the selected loudspeaker to execute the sonorous stimulus and register the position of the activated sensor.

#### Reaching of Auditory Cue Task

In order to test spatial perception in the auditory domain, we asked participants to reach a sonorous stimulus produced in turn by one out of the six target speakers (Figure 1B). The sonorous stimulus was a pink noise with a duration of 1 s. After the end of the stimulus, the participant moved the arm in order to place the handle over the sound source position and the experimenter confirmed his response by touching the corresponding position over the tactile surface. The six target positions were equally distributed in order to test auditory spatial perception on the entire workspace (target loudspeakers: 3, 5, 7, 10, 12, 14 with loudspeaker number one being the closest to the participants in each configuration). Each target was presented in randomized order for five times, for a total of 30 trials.

#### Joint Position Matching Task

In order to test spatial perception in the proprioceptive domain, we asked participants to perform an ipsilateral joint position matching task (Goble, 2010). After guiding the participants’ arm from the starting position corresponding to loudspeaker number 1 to the target proprioceptive position and then back to the starting position, the experimenter asked participants to replicate the movement in order to indicate the proprioceptive position experienced. Then the experimenter confirmed the participant’s response by touching the corresponding position over the tactile surface. The six target positions were the same as the auditory task. Each target was presented in randomized order for five times, for a total of 30 trials.

### Training Phase

Between the pre-test and post-test assessment phases, participants performed an audio-motor training that coupled the proprioceptive feedback and the auditory feedback from the body thanks to the use of a device that produces a sound whenever moved. The device is called Audio Bracelet for Blind Interaction (Finocchietti et al., 2015b) and it is a system developed to train spatial abilities in visually impaired people thanks to its potential to associate motor and auditory signals from the body (Finocchietti et al., 2015a; Cappagli et al., 2017b, 2019).

The training lasted 10 min in total, divided into four blocks of 2.5 min each. Between each training block, participants rested for 5 min. During the training, participants wore on the dominant arm the wearable audio device while their wrist was passively moved by the experimenter on the rail over the setup in two ways: (a) continuous back-and-forth movement along the setup; (b) discrete back-and-forth movements where the participants’ arm was positioned for 1 s over each of the sixteen loudspeakers embedded in the setup. The main aim of the training was to couple the proprioceptive feedback deriving from arm displacement with the auditory feedback deriving from the auditory source positioned on their wrist. The differentiation between continuous and discrete movements helped participants to respectively explore the setup and understand where each target position was placed by combining auditory and proprioceptive information. The ABBI was programmed in remote control, therefore, the audio command was triggered by the experimenter using a mobile phone. The wearable device produced a continuous pink noise sound.

### Analysis

In order to evaluate the accuracy and the precision of participants in both the *Reaching of Auditory Cue* and the *Joint Position Matching* tasks, we computed the distance error in millimeters between each target and indicated position and then averaged across all target positions, extracting two variables: Matching Error (ME) and the Variability (SD).

Matching Error represents a measure of accuracy or its inverse, bias. It is defined as the Euclidean distance between the target and the final arm position.

(1) $$M\,E=\sum_{i=1}^{N}{\left(x_{E\,E}-x_{T\,G}\right)}^{2}$$

where N is the number of Target repetitions (5), *x*_*EE*_ is the participants’ final position and *x*_*TG*_ is the Target position. This variable is then averaged across targets.

The Variability (SD) is a measure of precision and it is evaluated as the standard deviation of the error positions.

(2) $$S\,D=\sqrt{\frac{1}{N-1}\,\sum_{i=1}^{N}{\left(d_{i}-\underline{d}\right)}^{2}}$$

where d is the error distance *x*_*EE*_−*x*_*TG*_, and N is the number of target repetitions. SD is evaluated for each target and then averaged.

For both variables, we performed the incremental difference pre-post training (Δ), as follows:

(3) $$\Delta =100\,\frac{v\,a\,r_{p\,r\,e}-v\,a\,r_{p\,o\,s\,t}}{v\,a\,r_{p\,r\,e}}$$

where *var*_*pre*_ represents the performance at the pre-training assessment session and *var*_*post*_ represents the performance at the post-training assessment session.

## Results

### Proprioceptive and Auditory Spatial Representations

**FIGURE 2:**
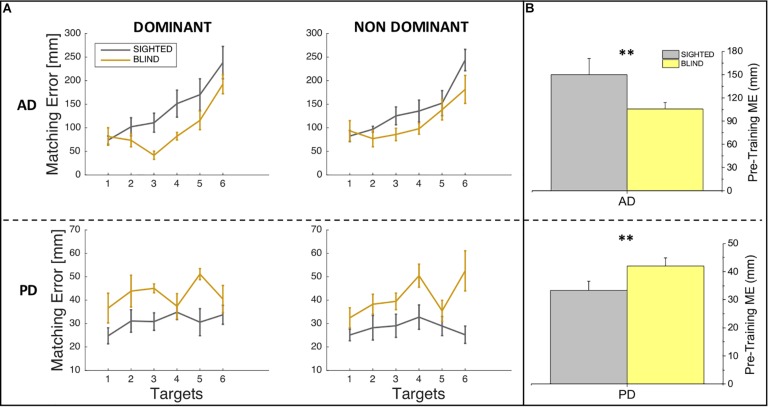
Auditory and proprioceptive performance at the pre-training session. **(A)** The panel represents the auditory (top) and proprioceptive (bottom) matching errors (in mm) for the dominant (left) and non-dominant (right) arms. Results are shown for each target location and indicate that for the auditory but not for the proprioceptive domain, matching error increases therefore performance decreases with increasing target location distance. **(B)** The panel represents the auditory (top) and proprioceptive (bottom) matching errors (in mm) independently of the arm trained (dominant, non-dominant). Results indicate that blind participants outperformed sighted participants in the auditory domain, while sighted participants outperformed blind participants in the proprioceptive domain. ^∗∗^Indicates *p*-values < 0.01.

In order to investigate whether sighted and blind individuals differ in their auditory and proprioceptive spatial representations, we compared the performance of sighted and blind participants at the pre-training session in auditory and proprioceptive domains separately both for ME and variability variables. Specifically, we performed four two-way ANOVAs with group (sighted, blind) and side (dominant, non-dominant) as main factors separately for auditory domain and proprioceptive domain and for ME and variability (SD). In case of significant effect (*p* < 0.05), we applied the *post hoc t*-test with Bonferroni correction. Figure 2A depicts auditory and proprioceptive spatial accuracy in terms of ME of sighted and blind participants at the pre-training session for the dominant and non-dominant arms for all six target locations, while Figure 2B depicts the auditory and proprioceptive spatial performance of sighted and blind participants independently of the arm considered (dominant, non-dominant) and across all target locations. The statistical analysis of spatial accuracy (ME) confirms what shown in Figure 2, which is that for the auditory domain a significant difference in terms of auditory accuracy exists between sighted and blind participants (*F* = 10.33, *p* = 0.003) while neither main effect of side (*F* = 0.03, *p* > 0.05) nor interaction between group and side (*F* = 0.62, *p* > 0.05) exist, suggesting that overall sighted individuals are less accurate than blind individuals for audio spatial localization [*t*(14) = 2.7, *p* = 0.015, Figure 2B, top panel]. Opposite results are shown for the proprioceptive domain, for which a significant difference in terms of proprioceptive accuracy exists between sighted and blind participants (*F* = 8.87, *p* = 0.005) while neither main SIDE effect (*F* = 0.25, *p* > 0.05) nor interaction between group and side (*F* = 0.2, *p* > 0.05) exist, suggesting overall that blind individuals are less accurate than sighted individuals for proprioceptive spatial localization [*t*(14) = −2.51, *p* = 0.024, Figure 2B, bottom panel].

**FIGURE 3:**
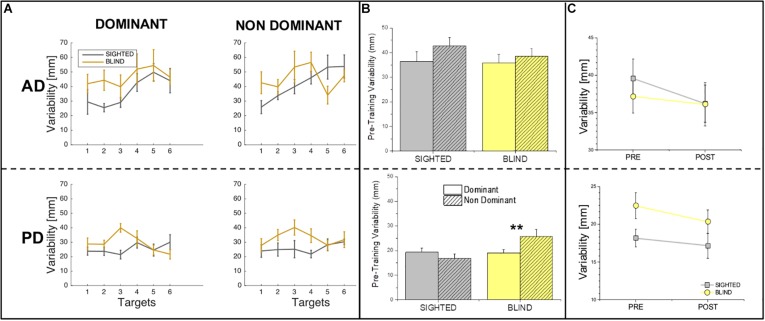
Variability in the auditory and proprioceptive domains. **(A)** The panel represents the auditory (top) and proprioceptive (bottom) variability (in mm) for the dominant (left) and non-dominant (right) arms. Results are shown for each target location and indicate that for both the auditory and proprioceptive domain, variability is target location independent for both groups. **(B)** The panel represents the auditory (top) and proprioceptive (bottom) variability (in mm) at the pre-training session for the dominant (plain bars) and non-dominant (pattern bars) arms. Results indicate that for each group, there is not difference in terms of variability between the dominant and non-dominant sides across domains with the only exception for blind participant in the Proprioceptive domain, who present a higher variability on the non-dominant hand. **(C)** The panel represents the comparison of variability (mm) in the pre-training and post-training sessions across sides (dominant and non-dominant pulled together) in the auditory (top) and proprioceptive (bottom). Results indicate that for both auditory and proprioceptive domains, variability does not change from the pre-training to the post-training session neither for the sighted nor for the blind participants. ^∗∗^Indicates *p*-values < 0.01.

Figure 3A depicts the difference between the performance of sighted and blind individuals at the pre-training session for auditory and proprioceptive spatial precision (SD) for all target locations, while Figure 3B shows the same comparison between sighted and blind participants across targets locations. The statistical analysis of SD revealed that in the auditory domain, no main effects of group (sighted vs. blind, *F* = 0.52, *p* > 0.05), side (dominant vs. non-dominant, *F* = 1.84, *p* > 0.05) or interaction (group × side, *F* = 0.31, *p* > 0.05) exist, suggesting overall that sighted individuals are as precise as blind individuals for audio spatial localization independently of the side of the body used to localize sounds (Figures 3A,B, top panel). Instead variability analysis in the proprioceptive domain reveals that both a significant difference between groups (*F* = 4.69, *p* = 0.039) and a significant interaction between group and side (*F* = 5.26, *p* = 0.029) exist while no main effect of side is present (*F* = 1.19, *p* > 0.05), suggesting that blind participants are less precise in the non-dominant compared to the dominant arm [*t*(8) = −3.5, *p* = 0.008, Figures 3A,B, bottom panel].

### Training Effect on Proprioceptive and Auditory Spatial Representations

In order to evaluate the effect of the audio-motor training on auditory and proprioceptive spatial representation, we performed two main analyses, respectively related to the ME and ΔME variables (see Analysis). Specifically, for ME we performed four three-way ANOVAs with group (sighted, blind), side (dominant, non-dominant) and time (pre, post) as main factors separately for auditory domain and proprioceptive domain and for ME and variability (SD). In case of significant effect (*p* < 0.05), we applied the *post hoc t*-test with Bonferroni correction. For ΔME we performed a two-way ANOVA with group (sighted, blind) and side (dominant, non-dominant) as main factors and the consequent *post hoc t*-test with Bonferroni correction in case of significant result.

**FIGURE 4:**
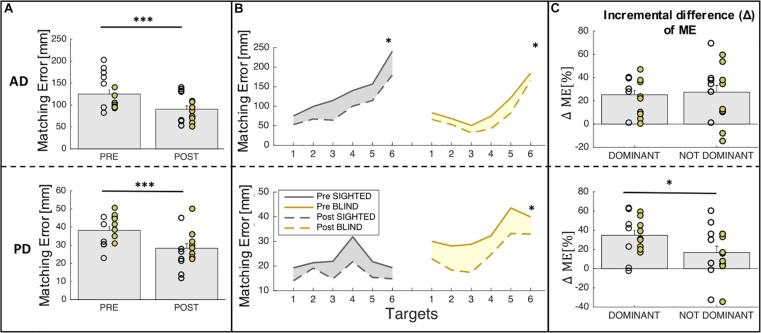
Auditory and proprioceptive performance after the training session. **(A)** The panel shows the mean (bars) and individual (circles) values of ME before and after training; yellow represent blind individuals while gray represent sighted individuals. Results indicate that matching error decreases after the training both for auditory and proprioceptive domain. **(B)** The panel represents the auditory (top) and proprioceptive (bottom) matching errors (in mm) independently on the body side (dominant and non-dominant). Results are shown for each target location in each domain. **(C)** The panel represents the auditory (top) and proprioceptive (bottom) Δ matching errors (ΔME in %) for the trained (dominant) and the not trained (non-dominant) sides in both groups (sighted, blind). Results indicate that for the proprioceptive domain, participants improve their spatial performance more in the dominant side on which they performed the training. ^∗∗∗^Indicates *p*-values < 0.001 and ^∗^ indicates *p*-values < 0.05.

Training results for ME and ΔME are shown in Figure 4. Figure 4A shows the mean ME of the pre-training and post-training phases for both groups (sighted and blind) across sides (dominant, non-dominant). Figure 4B depicts the auditory and proprioceptive spatial performance of sighted and blind participants at the pre-training (continuous line) and post-training (dashed line) sessions for all six target locations independently of side. Figure 4C depicts the ΔME expressed as incremental difference between the pre-training and post-training sessions for both groups (sighted and blind) for the dominant (trained) and the non-dominant (untrained) sides.

For what concerns the analysis related to spatial accuracy (ME), we found that for the auditory domain, there is a significant main effect of group (sighted vs. blind, *F* = 12.45, *p* = 0.0008) and time (pre vs. post, *F* = 14.27, *p* = 0.0004) but neither main effect of side (dominant vs. non-dominant, *F* = 0.1, *p* > 0.05) nor interactions among factors (group × time, *F* = 1.29, *p* > 0.05; group × side, *F* = 1.02, *p* > 0.05; time × side, *F* = 0.09, *p* > 0.05). Indeed Figure 4A (top panel) represents the main effect of time, for which participants (sighted and blind pooled together) decreased significantly their ME after training [*t*(15) = 6.7, *p* < 0.0001]. Moreover, as can be seen in Figure 4B (top panel), the ME decrease is homogeneous for all the target locations considered for both groups and when merging performance accuracy in the dominant and non-dominant arms both sighted [*t*(6) = 5.3, *p* = 0.002] and blind individuals [*t*(8) = 5.9, *p* = 0.0003] improved their performance. Similarly, for the proprioceptive domain, there is a significant main effect of group (sighted vs. blind, *F* = 11.61, *p* = 0.001) and time (pre vs. post, *F* = 17.16, *p* = 0.0001) but neither main effect of side (dominant vs. non-dominant, *F* = 0.48, *p* > 0.05) nor interactions among factors (group × time, *F* = 0.08, *p* > 0.05; group x side, *F* = 1.1, *p* > 0.05; time × side, *F* = 1.93, *p* > 0.05). Figure 4A (bottom panel) represents the main effect of time, for which participants (sighted and blind pooled together) decreased significantly their ME after training [*t*(15) = 5.76, *p* < 0.0001]. Moreover, as can be seen in Figure 4B (bottom panel), the ME decrease is homogeneous for all the target locations considered for both groups but when merging performance accuracy in the dominant and non-dominant arms, only blind individuals showed relevant enhancements in proprioceptive function after the training [*t*(8) = 4.9, *p* < 0.01] while sighted individuals showed a weaker improvement [*t*(6) = 3.08, *p* = 0.02 not significant with the Bonferroni correction >0.05].

For what concerns the analysis related to the incremental difference (Δ) of ME, which is the change of accuracy between pre and post training scaled by the initial error, we performed a two-way ANOVA with group (sighted, blind) and side (dominant, non-dominant) as main factors. The statistical analysis reported a significant side effect for the proprioceptive domain (*p* = 0.05) while neither main effect of group nor interaction between factors has been found. Figure 4C represents the main effect of side, for which the improvement after the training is equivalent for the dominant or trained side and the non-dominant or untrained side for the auditory domain (*p* > 0.05) but it is much higher for the dominant compared to the non-dominant side in the proprioceptive domain (dominant: 34.72% ± 5.12%; non-dominant: 16.74% ± 6.58%; *t*(15) = 2.7, *p* = 0.016).

For what concerns SD, the statistical analysis revealed that no effect of time on SD is present neither for the auditory domain (*F* = 0.7, *p* > 0.05) nor for the proprioceptive domain (*F* = 1.09, *p* > 0.05). Results for SD are depicted in Figure 3C by reporting the difference between the pre and post-training sessions for auditory and proprioceptive SD.

## Discussion

Despite the pivotal role of multisensory contingencies in the development of spatial perception, to date very few studies have investigated the effect of training based on audio-motor contingency on spatial competence in blind individuals. With this study, we demonstrated that training based on audio-motor contingencies enhances spatial perception of blind individuals in the auditory and proprioceptive domains, confirming the importance of sensory-motor experiences during therapeutic intervention.

This study highlights two main results. The first evidence is that after the audio-motor training, ME decreases in the trained (dominant) side for both sighted and blind individuals, while generalization effects much evident for the auditory domain in both groups. This result is in line with previous findings showing that auditory spatial perception in the blind can be enhanced with a proper training based on multisensory feedback (Aggius-Vella et al., 2017; Finocchietti et al., 2017; Cappagli et al., 2019) and that similarly both auditory and proprioceptive spatial capabilities can be improved in the sighted (Cuppone et al., 2018). The fact that a generalization effect to the untrained side of the body is more evident within the auditory domain for both sighted and blind participants can be due to the different nature of the auditory and proprioceptive modalities. Indeed, while audition is allocentric, proprioception is intrinsically egocentric therefore gains in spatial accuracy might not transfer as easily as within the auditory modality from a body part to another. The second result is that blind participants outperformed sighted participants in the auditory domain, while sighted participants outperformed blind participants in the proprioceptive domain in terms of spatial accuracy at the pre-training session. This result is in line with previous findings showing that proprioception can be altered in the blind (Rossetti et al., 1996; Gaunet and Rossetti, 2006; Cappagli et al., 2017a) but some aspects of auditory perception can be enhanced (Gori et al., 2013). Moreover, some evidence demonstrate that blindfolding procedures can alter perceptual capabilities in the sighted (Tabry et al., 2013). Finally, the fact that spatial accuracy decreases as target positions increases in both sighted and blind individuals suggests that similar perceptual mechanisms are in the act when auditory stimuli are processed, independently of overall performance accuracy.

The main aim of this study was to assess whether a training based on multisensory (audio-motor) feedback can improve spatial perception, more specifically can calibrate altered proprioceptive function. Participants were trained to couple the proprioceptive feedback deriving from arm displacement with the auditory feedback provided by the external source positioned on their wrist. On the contrary, most of the studies conducted so far have investigated the effect of more artificial training based on the use of sensory substitution devices. These approaches typically require to learn how to transform visual properties of a stimulus into auditory or tactile information. Specifically, for the blind, visual-to-auditory sensory substitution devices artificially translate visual properties of a stimulus into auditory information by means of specifically developed devices that mimic the physiological functions of the visual modality (Auvray and Myin, 2009; Velázquez, 2010). For example, in some cases, the information about the contrast between light and dark in a visual image is conveyed with sounds of different frequencies (Amedi et al., 2007). Sensory substitution devices can improve object localization (Renier et al., 2005) and form recognition (Arno et al., 1999; Cronly-Dillon et al., 1999; Cronly–Dillon et al., 2000; Pollok et al., 2005) by translating visual properties of surrounding objects via changes in auditory parameters such as pitch and amplitude. Nonetheless, it is worth noting that the perceptual outcome of such devices might result artificial in the sense that the auditory output provided by the system is not directly connected with the spatial information but strictly depends on the codification rules applied by the coupling system, which are typically internalized by users through extensive training. Moreover, we recently outlined that not all the technological devices developed so far can be used by blind individuals in their everyday life, principally due to the long and extensive training they require. For this reason, our aim was to test whether a simpler device that provides audio-motor contingencies can enhance auditory and proprioceptive functions in the blind adult.

In conclusion, we demonstrated that spatial perception can improve in blind individuals thanks to training based on audio-motor contingencies, confirming the importance of multisensory experiences to acquire spatial competence. Overall the findings of the present study confirmed the importance of visual experience in the construction and calibration of non-visual spatial maps and stressed the importance of early therapeutic intervention to support the acquisition of fundamental spatial competencies from infancy.

## Data Availability Statement

The datasets generated for this study are available on request to the corresponding author.

## Ethics Statement

The research conformed to the ethical standards laid down in the 1964 Declaration of Helsinki and was approved by the local ethics committee (ASL3 Ligure). Each participant signed a consent form conforming to these guidelines.

## Author Contributions

AC, GC, and MG developed the design of the study. AC collected most of the data. GC helped in the final part of data collection. AC and GC analyzed the data and wrote the manuscript. MG reviewed the manuscript and provided feedback for discussion of results.

## Conflict of Interest

The authors declare that the research was conducted in the absence of any commercial or financial relationships that could be construed as a potential conflict of interest.
